# Metacridamide B methanol-*d*_4_ monosolvate

**DOI:** 10.1107/S1600536813009641

**Published:** 2013-04-17

**Authors:** Ulrich Englich, Stuart B. Krasnoff

**Affiliations:** a Department of Chemistry, Syracuse University, 1-014 Center for Science & Technology, Syracuse, NY 13244-4100, USA; b USDA–ARS, Biological IPM Research Unit, Robt. W. Holley Ctr. for Ag. and Health, Tower Rd, Ithaca, NY 14853, USA

## Abstract

The title compound, C_35_H_53_NO_5_·CH_3_OH {systematic name: (3*S*,6*E*,8*S*,9*R*,10*E*,12*S*,13*S*,14*E*,16*S*,17*R*)-3-benzyl-9,13-dihy­droxy-6,8,10,12,14,16-hexa­methyl-17-[(2*S*,4*S*)-4-methyl­hexan-2-yl]-1-oxa-4-aza­cyclo­hepta­deca-6,10,14-triene-2,5-dione methanol-*d*_4_ monosolvate}, was extracted from conidia of the fungus *Metarhizium acridum*. Crystals were obtained as a methanol-*d*_4_ solvate. The tail part of the 4-methyl­hexan-2-yl group exhibits disorder over two positions, with an occupancy ratio of 0.682 (9):0.318 (9). The crystal structure confirms the absolute configuration of nine stereocenters determined previously for the acetyl­ated compound metacridamide A. In the crystal, the methanol-*d*4 mol­ecule is positioned close to the O atom in the carbonyl group of the peptide bond, forming an O—H⋯O hydrogen bond. It also forms an O—H⋯O hydrogen bond with an adjacent mol­ecule. N—H⋯O and O—H⋯O hydrogen bonds are observed between neighboring mol­ecules.

## Related literature

For details of the isolation and purification of the title compound, see: Krasnoff *et al.* (2012 ▶). 
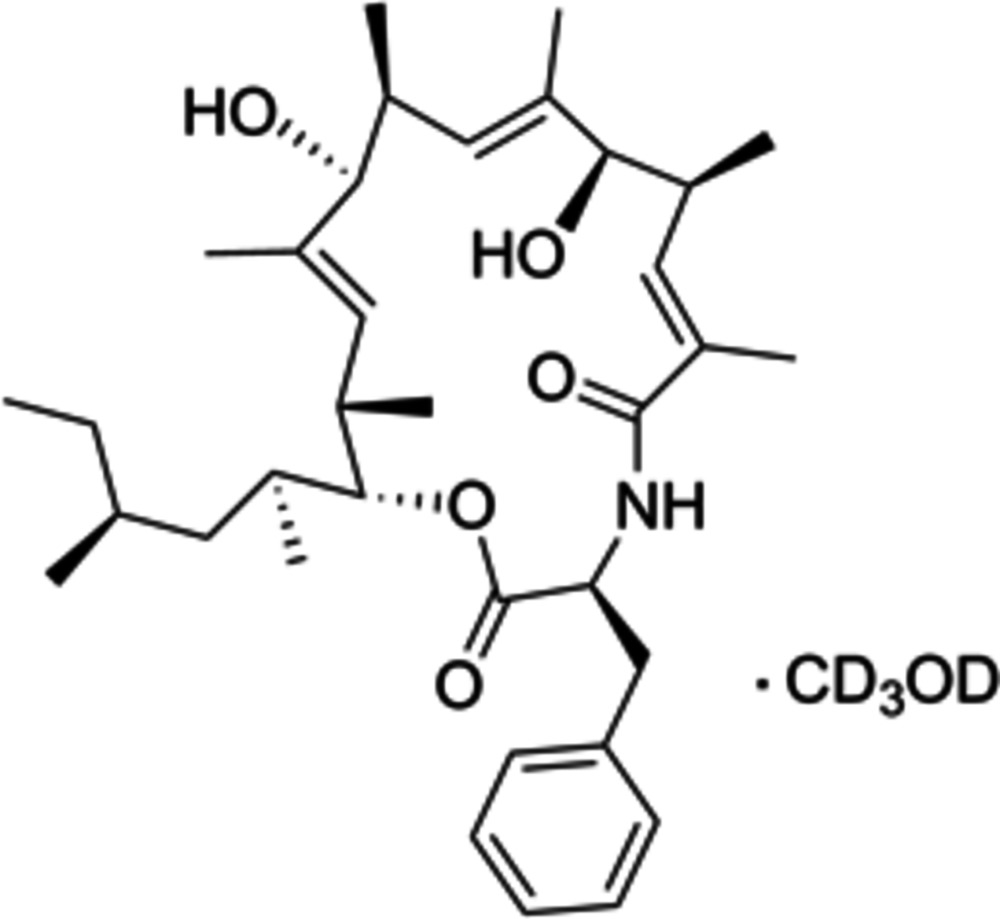


## Experimental

### 

#### Crystal data

C_35_H_53_NO_5_·CH_4_O
*M* *_r_* = 599.83
Orthorhombic, 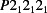

*a* = 8.5444 (3) Å
*b* = 10.9406 (4) Å
*c* = 37.8017 (15) Å
*V* = 3533.7 (2) Å^3^
*Z* = 4
Cu *K*α radiation
μ = 0.60 mm^−1^
*T* = 90 K
0.30 × 0.30 × 0.10 mm

#### Data collection

Bruker APEXII CCD diffractometer
Absorption correction: multi-scan (*SADABS*; Bruker, 2008 ▶) *T*_min_ = 0.841, *T*_max_ = 0.943
15638 measured reflections
6147 independent reflections
6037 reflections with *I* > 2σ(*I*)
*R*_int_ = 0.021

#### Refinement

*R*[*F* ^2^ > 2σ(*F* ^2^)] = 0.043
*wR*(*F* ^2^) = 0.115
*S* = 1.11
6147 reflections
397 parameters
H-atom parameters constrained
Δρ_max_ = 0.28 e Å^−3^
Δρ_min_ = −0.33 e Å^−3^
Absolute structure: Flack (1983 ▶), 2547 Friedel pairs
Flack parameter: 0.12 (18)

### 

Data collection: *APEX2* (Bruker, 2008 ▶); cell refinement: *SAINT* (Bruker, 2008 ▶); data reduction: *SAINT* (Bruker, 2008 ▶); program(s) used to solve structure: *SHELXS97* (Sheldrick, 2008 ▶); program(s) used to refine structure: *SHELXL97* (Sheldrick, 2008 ▶); molecular graphics: *Mercury* (Macrae *et al.*, 2006 ▶); software used to prepare material for publication: *SHELXTL* (Sheldrick, 2008 ▶).

## Supplementary Material

Crystal structure: contains datablock(s) I, global. DOI: 10.1107/S1600536813009641/fj2623sup1.cif

Structure factors: contains datablock(s) I. DOI: 10.1107/S1600536813009641/fj2623Isup2.hkl

Supplementary material file. DOI: 10.1107/S1600536813009641/fj2623Isup3.cml

Additional supplementary materials:  crystallographic information; 3D view; checkCIF report

## Figures and Tables

**Table 1:** Hydrogen-bond geometry (Å, °)

*D*—H⋯*A*	*D*—H	H⋯*A*	*D*⋯*A*	*D*—H⋯*A*
N4—H4*A*⋯O5^i^	0.88	2.16	2.915 (2)	144
O4—H4*B*⋯O1*S* ^ii^	0.84	2.00	2.795 (2)	157
O5—H5*A*⋯O2^iii^	0.84	2.06	2.839 (2)	154
O1*S*—H1*S*⋯O3	0.84	1.93	2.754 (2)	168

Symmetry codes: (i) 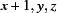; (ii) 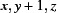; (iii) 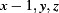.

**Fig. 1.**
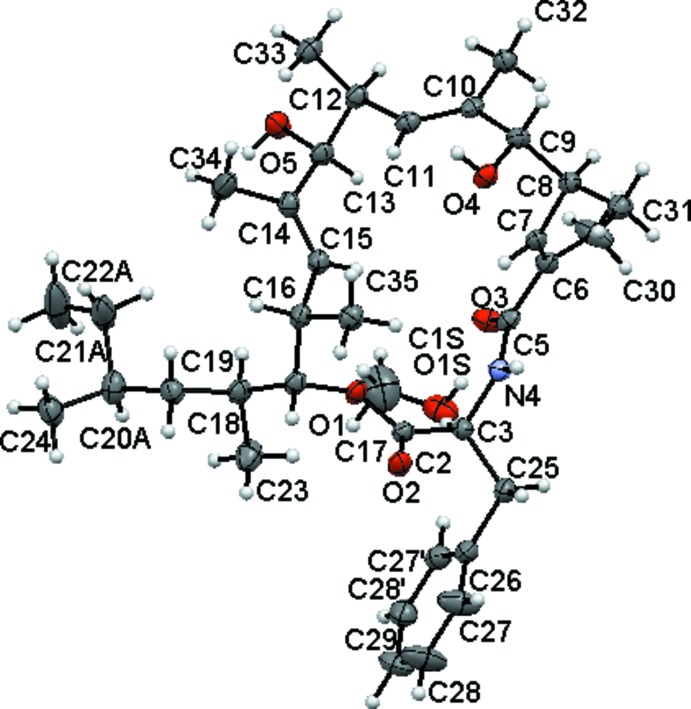
: The molecular structure of the title compound with displacement ellipsoids drawn at 50% probability level. The atoms of the minor disordered component C20B–C22B have been omitted for clarity.

## supplementary crystallographic information

### Refinement

The title compound crystallizes with one methanol solvent molecule in the asymmetric unit. The aliphatic sidechain C20 - C22 has been found to be disordered and therefore modeled with 2 parts A and B, keeping the C20A and C20B restrained in positional and displacement parameters, while C21A, C21B,and C22A, C22B resp. were restrained in their anisotropic displacement parameters. Occupancy has been refined to be 0.682 (9)/0.318 (9). All hydrogen atoms have been placed in idealized positions (riding model) with displacement parameters *U*_iso_ 1.2 times the values of the resp. carrier atom for CH and CH_2_ and 1.5 times the value of the carrier atom for CH_3_ and OH groups.

### Crystal data

**Table:**

C_35_H_53_NO_5_·CH_4_O	*F*(000) = 1312
*M**_r_* = 599.83	*D*_x_ = 1.127 Mg m^−^^3^
Orthorhombic, *P*2_1_2_1_2_1_	Cu *K*α radiation, λ = 1.54178 Å
*a* = 8.5444 (3) Å	θ = 4.2–67.8°
*b* = 10.9406 (4) Å	µ = 0.60 mm^−^^1^
*c* = 37.8017 (15) Å	*T* = 90 K
*V* = 3533.7 (2) Å^3^	Block, colourless
*Z* = 4	0.30 × 0.30 × 0.10 mm

### Data collection

**Table:**

Bruker APEXII CCD diffractometer	6147 independent reflections
Radiation source: microfocus sourse, Incoatec ImuS micro-focus sealed tube	6037 reflections with *I* > 2σ(*I*)
Incoatech ImuS multilayer optics monochromator	*R*_int_ = 0.021
φ and ω scans	θ_max_ = 68.2°, θ_min_ = 4.2°
Absorption correction: multi-scan (*SADABS*; Bruker, 2008)	*h* = −5→10
*T*_min_ = 0.841, *T*_max_ = 0.943	*k* = −13→12
15638 measured reflections	*l* = −41→45

### Refinement

**Table:**

Refinement on *F*^2^	Secondary atom site location: difference Fourier map
Least-squares matrix: full	Hydrogen site location: inferred from neighbouring sites
*R*[*F*^2^ > 2σ(*F*^2^)] = 0.043	H-atom parameters constrained
*wR*(*F*^2^) = 0.115	*w* = 1/[σ^2^(*F*_o_^2^) + (0.0564*P*)^2^ + 1.4912*P*] where *P* = (*F*_o_^2^ + 2*F*_c_^2^)/3
*S* = 1.11	(Δ/σ)_max_ < 0.001
6147 reflections	Δρ_max_ = 0.28 e Å^−^^3^
397 parameters	Δρ_min_ = −0.33 e Å^−^^3^
0 restraints	Absolute structure: Flack (1983), 2547 Friedel pairs
Primary atom site location: structure-invariant direct methods	Flack parameter: 0.12 (18)

### Special details

**Table:**

Geometry. All e.s.d.'s (except the e.s.d. in the dihedral angle between two l.s. planes) are estimated using the full covariance matrix. The cell e.s.d.'s are taken into account individually in the estimation of e.s.d.'s in distances, angles and torsion angles; correlations between e.s.d.'s in cell parameters are only used when they are defined by crystal symmetry. An approximate (isotropic) treatment of cell e.s.d.'s is used for estimating e.s.d.'s involving l.s. planes.
Refinement. Refinement of *F*^2^ against ALL reflections. The weighted *R*-factor *wR* and goodness of fit *S* are based on *F*^2^, conventional *R*-factors *R* are based on *F*, with *F* set to zero for negative *F*^2^. The threshold expression of *F*^2^ > σ(*F*^2^) is used only for calculating *R*-factors(gt) *etc*. and is not relevant to the choice of reflections for refinement. *R*-factors based on *F*^2^ are statistically about twice as large as those based on *F*, and *R*- factors based on ALL data will be even larger.

### Fractional atomic coordinates and isotropic or equivalent isotropic displacement parameters (Å^2^)

**Table:**

	*x*	*y*	*z*	*U*_iso_*/*U*_eq_	Occ. (<1)
O1	0.24840 (16)	0.71863 (13)	0.14560 (3)	0.0243 (3)	
C2	0.4004 (2)	0.70726 (18)	0.13849 (5)	0.0218 (4)	
O2	0.50540 (16)	0.75006 (13)	0.15593 (4)	0.0277 (3)	
C3	0.4207 (2)	0.63685 (18)	0.10401 (5)	0.0224 (4)	
H3A	0.3373	0.5729	0.1027	0.027*	
C25	0.5796 (2)	0.57359 (19)	0.10150 (5)	0.0268 (4)	
H25A	0.5875	0.5309	0.0785	0.032*	
H25B	0.6635	0.6359	0.1025	0.032*	
C26	0.6035 (2)	0.48209 (19)	0.13115 (5)	0.0263 (4)	
C27	0.7262 (3)	0.4954 (2)	0.15475 (8)	0.0491 (7)	
H27A	0.7964	0.5622	0.1522	0.059*	
C28	0.7478 (4)	0.4124 (3)	0.18209 (9)	0.0583 (8)	
H28A	0.8327	0.4225	0.1980	0.070*	
C29	0.6469 (3)	0.3158 (2)	0.18613 (6)	0.0413 (6)	
H29A	0.6621	0.2587	0.2048	0.050*	
C28'	0.5243 (3)	0.3021 (2)	0.16313 (6)	0.0355 (5)	
H28B	0.4538	0.2357	0.1659	0.043*	
C27'	0.5026 (3)	0.3848 (2)	0.13574 (6)	0.0306 (5)	
H27B	0.4172	0.3744	0.1199	0.037*	
N4	0.39658 (19)	0.72275 (15)	0.07506 (4)	0.0224 (3)	
H4A	0.4616	0.7846	0.0728	0.027*	
C5	0.2785 (2)	0.71150 (17)	0.05146 (5)	0.0225 (4)	
O3	0.18573 (18)	0.62545 (13)	0.05191 (4)	0.0315 (3)	
C6	0.2685 (2)	0.80822 (18)	0.02370 (5)	0.0242 (4)	
C30	0.2266 (4)	0.7595 (2)	−0.01239 (6)	0.0461 (7)	
H30A	0.2223	0.8272	−0.0293	0.069*	
H30B	0.1242	0.7192	−0.0113	0.069*	
H30C	0.3060	0.7004	−0.0200	0.069*	
C7	0.2905 (2)	0.92489 (18)	0.03213 (5)	0.0227 (4)	
H7A	0.3144	0.9419	0.0562	0.027*	
C8	0.2815 (2)	1.03320 (18)	0.00752 (5)	0.0230 (4)	
H8A	0.2302	1.0063	−0.0149	0.028*	
C31	0.4462 (3)	1.0795 (2)	−0.00130 (6)	0.0289 (5)	
H31A	0.5072	1.0132	−0.0119	0.043*	
H31B	0.4978	1.1072	0.0204	0.043*	
H31C	0.4388	1.1478	−0.0180	0.043*	
C9	0.1814 (2)	1.13529 (18)	0.02408 (5)	0.0231 (4)	
H9A	0.1819	1.2066	0.0075	0.028*	
O4	0.25721 (16)	1.17147 (13)	0.05594 (4)	0.0254 (3)	
H4B	0.2052	1.2272	0.0657	0.038*	
C10	0.0131 (2)	1.09516 (17)	0.02921 (5)	0.0220 (4)	
C32	−0.0729 (2)	1.0701 (2)	−0.00471 (5)	0.0266 (4)	
H32A	−0.1803	1.0446	0.0006	0.040*	
H32B	−0.0194	1.0049	−0.0177	0.040*	
H32C	−0.0751	1.1445	−0.0191	0.040*	
C11	−0.0488 (2)	1.08391 (18)	0.06150 (5)	0.0240 (4)	
H11A	0.0182	1.1026	0.0808	0.029*	
C12	−0.2128 (2)	1.04501 (19)	0.07077 (5)	0.0249 (4)	
H12A	−0.2648	1.0195	0.0483	0.030*	
C33	−0.3060 (3)	1.1537 (2)	0.08555 (6)	0.0319 (5)	
H33A	−0.3038	1.2212	0.0685	0.048*	
H33B	−0.2591	1.1806	0.1079	0.048*	
H33C	−0.4146	1.1288	0.0897	0.048*	
C13	−0.2092 (2)	0.93239 (19)	0.09515 (5)	0.0251 (4)	
H13A	−0.1563	0.8653	0.0818	0.030*	
O5	−0.36908 (17)	0.89529 (14)	0.10053 (4)	0.0311 (3)	
H5A	−0.3753	0.8528	0.1190	0.047*	
C14	−0.1219 (2)	0.94720 (18)	0.12989 (5)	0.0249 (4)	
C34	−0.1984 (3)	1.0210 (3)	0.15900 (6)	0.0426 (6)	
H34A	−0.1298	1.0225	0.1798	0.064*	
H34B	−0.2987	0.9835	0.1654	0.064*	
H34C	−0.2162	1.1047	0.1507	0.064*	
C15	0.0193 (2)	0.89721 (18)	0.13276 (5)	0.0247 (4)	
H15A	0.0501	0.8451	0.1139	0.030*	
C16	0.1368 (2)	0.91269 (19)	0.16215 (5)	0.0248 (4)	
H16A	0.0849	0.9574	0.1820	0.030*	
C35	0.2724 (3)	0.99202 (19)	0.14830 (6)	0.0291 (4)	
H35A	0.3502	1.0030	0.1671	0.044*	
H35B	0.2319	1.0720	0.1410	0.044*	
H35C	0.3213	0.9516	0.1280	0.044*	
C17	0.1972 (2)	0.79026 (18)	0.17640 (5)	0.0237 (4)	
H17A	0.2887	0.8053	0.1923	0.028*	
C18	0.0781 (2)	0.7101 (2)	0.19556 (5)	0.0269 (4)	
H18A	−0.0165	0.7011	0.1802	0.032*	
C23	0.1484 (3)	0.5832 (2)	0.20208 (7)	0.0404 (6)	
H23A	0.1801	0.5471	0.1795	0.061*	
H23B	0.0701	0.5308	0.2134	0.061*	
H23C	0.2399	0.5907	0.2176	0.061*	
C19	0.0280 (3)	0.7675 (2)	0.23088 (5)	0.0291 (5)	
H19A	0.1186	0.7664	0.2472	0.035*	
H19B	0.0000	0.8541	0.2267	0.035*	
C20A	−0.1103 (3)	0.7044 (2)	0.24910 (6)	0.0365 (5)	0.682 (9)
H20A	−0.1031	0.6138	0.2459	0.044*	0.682 (9)
C21A	−0.2660 (7)	0.7548 (8)	0.23126 (16)	0.0597 (19)	0.682 (9)
H21A	−0.2845	0.8397	0.2393	0.072*	0.682 (9)
H21B	−0.2524	0.7564	0.2053	0.072*	0.682 (9)
C22A	−0.4090 (6)	0.6764 (9)	0.24045 (15)	0.081 (3)	0.682 (9)
H22A	−0.5025	0.7115	0.2294	0.121*	0.682 (9)
H22B	−0.4229	0.6745	0.2662	0.121*	0.682 (9)
H22C	−0.3930	0.5930	0.2317	0.121*	0.682 (9)
C20B	−0.1103 (3)	0.7044 (2)	0.24910 (6)	0.0365 (5)	0.318 (9)
H20B	−0.0716	0.6182	0.2502	0.044*	0.318 (9)
C21B	−0.2570 (18)	0.6875 (16)	0.2317 (4)	0.0597 (19)	0.318 (9)
H21C	−0.2353	0.6595	0.2073	0.072*	0.318 (9)
H21D	−0.3082	0.7684	0.2299	0.072*	0.318 (9)
C22B	−0.3761 (15)	0.598 (2)	0.2483 (3)	0.081 (3)	0.318 (9)
H22D	−0.4735	0.5996	0.2346	0.121*	0.318 (9)
H22E	−0.3978	0.6223	0.2727	0.121*	0.318 (9)
H22F	−0.3327	0.5150	0.2480	0.121*	0.318 (9)
C24	−0.1175 (3)	0.7359 (2)	0.28804 (6)	0.0387 (6)	
H24A	−0.2082	0.6956	0.2988	0.058*	
H24B	−0.1274	0.8247	0.2908	0.058*	
H24C	−0.0216	0.7079	0.2997	0.058*	
O1S	0.1574 (2)	0.39740 (14)	0.08205 (4)	0.0401 (4)	
H1S	0.1714	0.4614	0.0702	0.060*	
C1S	0.0341 (5)	0.4107 (3)	0.10224 (10)	0.0731 (10)	
H1S1	0.0390	0.3522	0.1218	0.110*	
H1S2	−0.0607	0.3958	0.0883	0.110*	
H1S3	0.0316	0.4941	0.1117	0.110*	

### Atomic displacement parameters (Å^2^)

**Table:**

	*U*^11^	*U*^22^	*U*^33^	*U*^12^	*U*^13^	*U*^23^
O1	0.0167 (7)	0.0315 (7)	0.0248 (6)	0.0010 (6)	0.0008 (5)	−0.0037 (6)
C2	0.0168 (9)	0.0244 (9)	0.0241 (9)	0.0012 (8)	0.0000 (8)	0.0032 (8)
O2	0.0186 (7)	0.0381 (8)	0.0264 (7)	−0.0016 (6)	−0.0009 (6)	−0.0002 (6)
C3	0.0204 (10)	0.0239 (9)	0.0228 (9)	0.0016 (8)	0.0002 (8)	0.0035 (8)
C25	0.0226 (10)	0.0300 (10)	0.0278 (10)	0.0017 (9)	0.0041 (8)	0.0030 (8)
C26	0.0217 (10)	0.0288 (10)	0.0284 (10)	0.0048 (8)	0.0028 (8)	0.0016 (8)
C27	0.0400 (15)	0.0413 (13)	0.0661 (17)	−0.0103 (12)	−0.0229 (13)	0.0200 (12)
C28	0.0549 (18)	0.0533 (16)	0.0666 (18)	−0.0072 (14)	−0.0357 (16)	0.0187 (14)
C29	0.0524 (16)	0.0339 (12)	0.0378 (12)	0.0042 (11)	−0.0116 (11)	0.0112 (10)
C28'	0.0372 (13)	0.0321 (11)	0.0374 (11)	0.0027 (10)	0.0026 (10)	0.0042 (9)
C27'	0.0264 (11)	0.0323 (11)	0.0330 (10)	0.0047 (9)	−0.0037 (9)	0.0005 (9)
N4	0.0200 (8)	0.0241 (8)	0.0231 (8)	−0.0014 (7)	−0.0009 (6)	0.0037 (7)
C5	0.0205 (9)	0.0211 (9)	0.0260 (9)	0.0024 (8)	−0.0002 (8)	−0.0049 (7)
O3	0.0353 (8)	0.0255 (7)	0.0337 (8)	−0.0039 (7)	−0.0108 (7)	0.0016 (6)
C6	0.0233 (10)	0.0275 (10)	0.0217 (9)	0.0024 (8)	−0.0027 (8)	−0.0016 (7)
C30	0.080 (2)	0.0273 (11)	0.0307 (11)	0.0019 (13)	−0.0210 (12)	0.0005 (9)
C7	0.0170 (9)	0.0293 (10)	0.0218 (9)	0.0022 (8)	−0.0007 (7)	0.0010 (8)
C8	0.0211 (10)	0.0253 (10)	0.0226 (9)	0.0007 (8)	−0.0009 (8)	0.0015 (7)
C31	0.0252 (11)	0.0290 (11)	0.0326 (11)	0.0013 (9)	0.0032 (9)	0.0039 (9)
C9	0.0204 (10)	0.0241 (9)	0.0247 (9)	0.0019 (8)	−0.0022 (8)	0.0011 (8)
O4	0.0195 (7)	0.0280 (7)	0.0287 (7)	−0.0010 (6)	−0.0011 (6)	−0.0040 (5)
C10	0.0196 (10)	0.0192 (9)	0.0272 (10)	0.0033 (7)	−0.0006 (8)	0.0004 (7)
C32	0.0216 (10)	0.0321 (11)	0.0262 (10)	−0.0015 (9)	−0.0020 (8)	0.0013 (8)
C11	0.0208 (10)	0.0239 (10)	0.0275 (10)	0.0001 (8)	−0.0021 (8)	−0.0009 (8)
C12	0.0193 (10)	0.0286 (10)	0.0269 (10)	0.0000 (8)	0.0005 (8)	−0.0013 (8)
C33	0.0227 (11)	0.0326 (11)	0.0405 (12)	0.0020 (9)	0.0034 (9)	−0.0010 (9)
C13	0.0174 (10)	0.0312 (10)	0.0267 (10)	−0.0023 (8)	−0.0003 (8)	−0.0008 (8)
O5	0.0210 (7)	0.0412 (8)	0.0309 (7)	−0.0090 (6)	−0.0016 (6)	0.0067 (7)
C14	0.0221 (10)	0.0287 (10)	0.0240 (10)	−0.0033 (8)	0.0022 (8)	−0.0014 (8)
C34	0.0277 (12)	0.0697 (17)	0.0305 (11)	0.0160 (12)	−0.0036 (10)	−0.0143 (11)
C15	0.0234 (10)	0.0279 (10)	0.0229 (9)	−0.0011 (8)	0.0018 (8)	−0.0018 (8)
C16	0.0215 (10)	0.0284 (10)	0.0245 (9)	0.0004 (8)	0.0026 (8)	−0.0032 (8)
C35	0.0245 (11)	0.0302 (10)	0.0325 (11)	−0.0026 (9)	0.0011 (9)	−0.0006 (8)
C17	0.0188 (9)	0.0292 (10)	0.0230 (9)	−0.0001 (8)	−0.0001 (8)	−0.0045 (8)
C18	0.0194 (9)	0.0342 (11)	0.0270 (10)	−0.0010 (9)	0.0000 (8)	−0.0012 (9)
C23	0.0408 (13)	0.0316 (12)	0.0488 (14)	0.0002 (10)	0.0149 (11)	0.0047 (11)
C19	0.0296 (11)	0.0323 (11)	0.0253 (10)	−0.0021 (9)	0.0019 (9)	0.0025 (9)
C20A	0.0327 (12)	0.0436 (13)	0.0332 (11)	−0.0074 (11)	0.0069 (10)	−0.0054 (10)
C21A	0.0262 (16)	0.121 (6)	0.0316 (14)	0.002 (4)	0.0036 (12)	0.010 (4)
C22A	0.033 (3)	0.173 (8)	0.036 (3)	−0.026 (4)	0.0055 (19)	0.001 (3)
C20B	0.0327 (12)	0.0436 (13)	0.0332 (11)	−0.0074 (11)	0.0069 (10)	−0.0054 (10)
C21B	0.0262 (16)	0.121 (6)	0.0316 (14)	0.002 (4)	0.0036 (12)	0.010 (4)
C22B	0.033 (3)	0.173 (8)	0.036 (3)	−0.026 (4)	0.0055 (19)	0.001 (3)
C24	0.0389 (13)	0.0482 (14)	0.0291 (11)	−0.0062 (11)	0.0055 (10)	0.0061 (10)
O1S	0.0545 (11)	0.0262 (8)	0.0396 (9)	−0.0006 (8)	−0.0109 (8)	0.0003 (7)
C1S	0.088 (3)	0.062 (2)	0.069 (2)	−0.0158 (19)	0.008 (2)	−0.0059 (17)

### Geometric parameters (Å, º)

**Table:**

O1—C2	1.332 (2)	C33—H33B	0.9800
O1—C17	1.470 (2)	C33—H33C	0.9800
C2—O2	1.208 (2)	C13—O5	1.440 (2)
C2—C3	1.524 (3)	C13—C14	1.519 (3)
C3—N4	1.457 (2)	C13—H13A	1.0000
C3—C25	1.527 (3)	O5—H5A	0.8400
C3—H3A	1.0000	C14—C15	1.330 (3)
C25—C26	1.517 (3)	C14—C34	1.513 (3)
C25—H25A	0.9900	C34—H34A	0.9800
C25—H25B	0.9900	C34—H34B	0.9800
C26—C27'	1.381 (3)	C34—H34C	0.9800
C26—C27	1.384 (3)	C15—C16	1.507 (3)
C27—C28	1.388 (4)	C15—H15A	0.9500
C27—H27A	0.9500	C16—C17	1.533 (3)
C28—C29	1.372 (4)	C16—C35	1.539 (3)
C28—H28A	0.9500	C16—H16A	1.0000
C29—C28'	1.370 (4)	C35—H35A	0.9800
C29—H29A	0.9500	C35—H35B	0.9800
C28'—C27'	1.387 (3)	C35—H35C	0.9800
C28'—H28B	0.9500	C17—C18	1.526 (3)
C27'—H27B	0.9500	C17—H17A	1.0000
N4—C5	1.352 (3)	C18—C23	1.533 (3)
N4—H4A	0.8800	C18—C19	1.536 (3)
C5—O3	1.231 (2)	C18—H18A	1.0000
C5—C6	1.493 (3)	C23—H23A	0.9800
C6—C7	1.329 (3)	C23—H23B	0.9800
C6—C30	1.508 (3)	C23—H23C	0.9800
C30—H30A	0.9800	C19—C20A	1.532 (3)
C30—H30B	0.9800	C19—H19A	0.9900
C30—H30C	0.9800	C19—H19B	0.9900
C7—C8	1.509 (3)	C20A—C24	1.513 (3)
C7—H7A	0.9500	C20A—C21A	1.590 (7)
C8—C31	1.532 (3)	C20A—H20A	1.0000
C8—C9	1.540 (3)	C21A—C22A	1.533 (8)
C8—H8A	1.0000	C21A—H21A	0.9900
C31—H31A	0.9800	C21A—H21B	0.9900
C31—H31B	0.9800	C22A—H22A	0.9800
C31—H31C	0.9800	C22A—H22B	0.9800
C9—O4	1.424 (2)	C22A—H22C	0.9800
C9—C10	1.516 (3)	C21B—C22B	1.54 (2)
C9—H9A	1.0000	C21B—H21C	0.9900
O4—H4B	0.8400	C21B—H21D	0.9900
C10—C11	1.336 (3)	C22B—H22D	0.9800
C10—C32	1.503 (3)	C22B—H22E	0.9800
C32—H32A	0.9800	C22B—H22F	0.9800
C32—H32B	0.9800	C24—H24A	0.9800
C32—H32C	0.9800	C24—H24B	0.9800
C11—C12	1.505 (3)	C24—H24C	0.9800
C11—H11A	0.9500	O1S—C1S	1.309 (4)
C12—C33	1.537 (3)	O1S—H1S	0.8400
C12—C13	1.539 (3)	C1S—H1S1	0.9800
C12—H12A	1.0000	C1S—H1S2	0.9800
C33—H33A	0.9800	C1S—H1S3	0.9800
			
C2—O1—C17	119.99 (15)	O5—C13—C14	111.96 (16)
O2—C2—O1	125.29 (18)	O5—C13—C12	106.95 (16)
O2—C2—C3	125.32 (17)	C14—C13—C12	116.25 (17)
O1—C2—C3	109.34 (16)	O5—C13—H13A	107.1
N4—C3—C2	107.46 (15)	C14—C13—H13A	107.1
N4—C3—C25	111.81 (16)	C12—C13—H13A	107.1
C2—C3—C25	112.58 (16)	C13—O5—H5A	109.5
N4—C3—H3A	108.3	C15—C14—C34	123.51 (19)
C2—C3—H3A	108.3	C15—C14—C13	118.19 (18)
C25—C3—H3A	108.3	C34—C14—C13	118.28 (18)
C26—C25—C3	111.91 (16)	C14—C34—H34A	109.5
C26—C25—H25A	109.2	C14—C34—H34B	109.5
C3—C25—H25A	109.2	H34A—C34—H34B	109.5
C26—C25—H25B	109.2	C14—C34—H34C	109.5
C3—C25—H25B	109.2	H34A—C34—H34C	109.5
H25A—C25—H25B	107.9	H34B—C34—H34C	109.5
C27'—C26—C27	118.2 (2)	C14—C15—C16	128.22 (19)
C27'—C26—C25	121.17 (19)	C14—C15—H15A	115.9
C27—C26—C25	120.6 (2)	C16—C15—H15A	115.9
C26—C27—C28	120.7 (2)	C15—C16—C17	112.65 (17)
C26—C27—H27A	119.6	C15—C16—C35	108.30 (17)
C28—C27—H27A	119.6	C17—C16—C35	111.02 (17)
C29—C28—C27	120.2 (2)	C15—C16—H16A	108.2
C29—C28—H28A	119.9	C17—C16—H16A	108.2
C27—C28—H28A	119.9	C35—C16—H16A	108.2
C28'—C29—C28	119.6 (2)	C16—C35—H35A	109.5
C28'—C29—H29A	120.2	C16—C35—H35B	109.5
C28—C29—H29A	120.2	H35A—C35—H35B	109.5
C29—C28'—C27'	120.3 (2)	C16—C35—H35C	109.5
C29—C28'—H28B	119.8	H35A—C35—H35C	109.5
C27'—C28'—H28B	119.8	H35B—C35—H35C	109.5
C26—C27'—C28'	120.9 (2)	O1—C17—C18	105.55 (16)
C26—C27'—H27B	119.6	O1—C17—C16	106.71 (15)
C28'—C27'—H27B	119.6	C18—C17—C16	116.37 (17)
C5—N4—C3	122.85 (17)	O1—C17—H17A	109.3
C5—N4—H4A	118.6	C18—C17—H17A	109.3
C3—N4—H4A	118.6	C16—C17—H17A	109.3
O3—C5—N4	122.75 (18)	C17—C18—C23	109.59 (17)
O3—C5—C6	120.98 (17)	C17—C18—C19	111.32 (17)
N4—C5—C6	116.25 (17)	C23—C18—C19	109.85 (18)
C7—C6—C5	120.27 (17)	C17—C18—H18A	108.7
C7—C6—C30	126.17 (19)	C23—C18—H18A	108.7
C5—C6—C30	113.52 (17)	C19—C18—H18A	108.7
C6—C30—H30A	109.5	C18—C23—H23A	109.5
C6—C30—H30B	109.5	C18—C23—H23B	109.5
H30A—C30—H30B	109.5	H23A—C23—H23B	109.5
C6—C30—H30C	109.5	C18—C23—H23C	109.5
H30A—C30—H30C	109.5	H23A—C23—H23C	109.5
H30B—C30—H30C	109.5	H23B—C23—H23C	109.5
C6—C7—C8	126.82 (18)	C20A—C19—C18	114.92 (18)
C6—C7—H7A	116.6	C20A—C19—H19A	108.5
C8—C7—H7A	116.6	C18—C19—H19A	108.5
C7—C8—C31	110.32 (16)	C20A—C19—H19B	108.5
C7—C8—C9	110.32 (16)	C18—C19—H19B	108.5
C31—C8—C9	111.02 (17)	H19A—C19—H19B	107.5
C7—C8—H8A	108.4	C24—C20A—C19	111.44 (19)
C31—C8—H8A	108.4	C24—C20A—C21A	107.4 (3)
C9—C8—H8A	108.4	C19—C20A—C21A	107.3 (3)
C8—C31—H31A	109.5	C24—C20A—H20A	110.2
C8—C31—H31B	109.5	C19—C20A—H20A	110.2
H31A—C31—H31B	109.5	C21A—C20A—H20A	110.2
C8—C31—H31C	109.5	C22A—C21A—C20A	112.1 (4)
H31A—C31—H31C	109.5	C22A—C21A—H21A	109.2
H31B—C31—H31C	109.5	C20A—C21A—H21A	109.2
O4—C9—C10	113.82 (16)	C22A—C21A—H21B	109.2
O4—C9—C8	107.03 (15)	C20A—C21A—H21B	109.2
C10—C9—C8	111.65 (16)	H21A—C21A—H21B	107.9
O4—C9—H9A	108.1	C21A—C22A—H22A	109.5
C10—C9—H9A	108.1	C21A—C22A—H22B	109.5
C8—C9—H9A	108.1	H22A—C22A—H22B	109.5
C9—O4—H4B	109.5	C21A—C22A—H22C	109.5
C11—C10—C32	124.70 (19)	H22A—C22A—H22C	109.5
C11—C10—C9	121.26 (18)	H22B—C22A—H22C	109.5
C32—C10—C9	114.03 (16)	C22B—C21B—H21C	107.7
C10—C32—H32A	109.5	C22B—C21B—H21D	107.7
C10—C32—H32B	109.5	H21C—C21B—H21D	107.1
H32A—C32—H32B	109.5	C21B—C22B—H22D	109.5
C10—C32—H32C	109.5	C21B—C22B—H22E	109.5
H32A—C32—H32C	109.5	H22D—C22B—H22E	109.5
H32B—C32—H32C	109.5	C21B—C22B—H22F	109.5
C10—C11—C12	127.37 (19)	H22D—C22B—H22F	109.5
C10—C11—H11A	116.3	H22E—C22B—H22F	109.5
C12—C11—H11A	116.3	C20A—C24—H24A	109.5
C11—C12—C33	110.38 (17)	C20A—C24—H24B	109.5
C11—C12—C13	110.32 (16)	H24A—C24—H24B	109.5
C33—C12—C13	114.33 (17)	C20A—C24—H24C	109.5
C11—C12—H12A	107.2	H24A—C24—H24C	109.5
C33—C12—H12A	107.2	H24B—C24—H24C	109.5
C13—C12—H12A	107.2	C1S—O1S—H1S	109.5
C12—C33—H33A	109.5	O1S—C1S—H1S1	109.5
C12—C33—H33B	109.5	O1S—C1S—H1S2	109.5
H33A—C33—H33B	109.5	H1S1—C1S—H1S2	109.5
C12—C33—H33C	109.5	O1S—C1S—H1S3	109.5
H33A—C33—H33C	109.5	H1S1—C1S—H1S3	109.5
H33B—C33—H33C	109.5	H1S2—C1S—H1S3	109.5
			
C17—O1—C2—O2	1.4 (3)	C8—C9—C10—C11	114.8 (2)
C17—O1—C2—C3	−176.01 (15)	O4—C9—C10—C32	173.62 (16)
O2—C2—C3—N4	−96.8 (2)	C8—C9—C10—C32	−65.1 (2)
O1—C2—C3—N4	80.66 (19)	C32—C10—C11—C12	0.0 (3)
O2—C2—C3—C25	26.8 (3)	C9—C10—C11—C12	−179.84 (18)
O1—C2—C3—C25	−155.80 (16)	C10—C11—C12—C33	−110.0 (2)
N4—C3—C25—C26	−178.74 (17)	C10—C11—C12—C13	122.7 (2)
C2—C3—C25—C26	60.2 (2)	C11—C12—C13—O5	−175.62 (16)
C3—C25—C26—C27'	59.2 (3)	C33—C12—C13—O5	59.3 (2)
C3—C25—C26—C27	−119.7 (2)	C11—C12—C13—C14	58.5 (2)
C27'—C26—C27—C28	0.6 (4)	C33—C12—C13—C14	−66.6 (2)
C25—C26—C27—C28	179.5 (3)	O5—C13—C14—C15	132.99 (19)
C26—C27—C28—C29	−0.2 (5)	C12—C13—C14—C15	−103.7 (2)
C27—C28—C29—C28'	−0.3 (5)	O5—C13—C14—C34	−48.7 (3)
C28—C29—C28'—C27'	0.4 (4)	C12—C13—C14—C34	74.6 (3)
C27—C26—C27'—C28'	−0.5 (3)	C34—C14—C15—C16	−7.0 (4)
C25—C26—C27'—C28'	−179.38 (19)	C13—C14—C15—C16	171.19 (19)
C29—C28'—C27'—C26	0.0 (4)	C14—C15—C16—C17	128.9 (2)
C2—C3—N4—C5	−116.88 (19)	C14—C15—C16—C35	−107.9 (2)
C25—C3—N4—C5	119.1 (2)	C2—O1—C17—C18	−131.99 (18)
C3—N4—C5—O3	−2.6 (3)	C2—O1—C17—C16	103.61 (19)
C3—N4—C5—C6	179.08 (17)	C15—C16—C17—O1	51.0 (2)
O3—C5—C6—C7	139.5 (2)	C35—C16—C17—O1	−70.67 (19)
N4—C5—C6—C7	−42.2 (3)	C15—C16—C17—C18	−66.5 (2)
O3—C5—C6—C30	−38.4 (3)	C35—C16—C17—C18	171.86 (17)
N4—C5—C6—C30	140.0 (2)	O1—C17—C18—C23	51.7 (2)
C5—C6—C7—C8	−178.82 (18)	C16—C17—C18—C23	169.85 (18)
C30—C6—C7—C8	−1.3 (4)	O1—C17—C18—C19	173.47 (16)
C6—C7—C8—C31	−104.4 (2)	C16—C17—C18—C19	−68.4 (2)
C6—C7—C8—C9	132.5 (2)	C17—C18—C19—C20A	170.56 (19)
C7—C8—C9—O4	61.5 (2)	C23—C18—C19—C20A	−67.9 (2)
C31—C8—C9—O4	−61.1 (2)	C18—C19—C20A—C24	160.6 (2)
C7—C8—C9—C10	−63.7 (2)	C18—C19—C20A—C21A	−82.1 (4)
C31—C8—C9—C10	173.69 (16)	C24—C20A—C21A—C22A	−74.4 (5)
O4—C9—C10—C11	−6.5 (3)	C19—C20A—C21A—C22A	165.6 (4)

### Hydrogen-bond geometry (Å, º)

**Table:**

*D*—H···*A*	*D*—H	H···*A*	*D*···*A*	*D*—H···*A*
N4—H4*A*···O5^i^	0.88	2.16	2.915 (2)	144
O4—H4*B*···O1*S*^ii^	0.84	2.00	2.795 (2)	157
O5—H5*A*···O2^iii^	0.84	2.06	2.839 (2)	154
O1*S*—H1*S*···O3	0.84	1.93	2.754 (2)	168

Symmetry codes: (i) *x*+1, *y*, *z*; (ii) *x*, *y*+1, *z*; (iii) *x*−1, *y*, *z*.

## References

[bb1] Bruker (2008). *APEX2*, *SAINT* and *SADABS* Bruker AXS Inc., Madison, Wisconsin, USA.

[bb2] Flack, H. D. (1983). *Acta Cryst.* A**39**, 876–881.

[bb3] Krasnoff, S. B., Englich, U., Miller, P. G., Shuler, M. L., Glahn, R. P., Donzelli, B. G. G. & Gibson, D. M. (2012). *J. Nat. Prod.* **75**(2), 175–180. 10.1021/np2007044 PMC3293398 22292922

[bb4] Macrae, C. F., Edgington, P. R., McCabe, P., Pidcock, E., Shields, G. P., Taylor, R., Towler, M. & van de Streek, J. (2006). *J. Appl. Cryst.* **39**, 453–457.

[bb5] Sheldrick, G. M. (2008). *Acta Cryst.* A**64**, 112–122. 10.1107/S0108767307043930 18156677

